# Plasma Based Markers of [^11^C] PiB-PET Brain Amyloid Burden

**DOI:** 10.1371/journal.pone.0044260

**Published:** 2012-09-24

**Authors:** Steven John Kiddle, Madhav Thambisetty, Andrew Simmons, Joanna Riddoch-Contreras, Abdul Hye, Eric Westman, Ian Pike, Malcolm Ward, Caroline Johnston, Michelle Katharine Lupton, Katie Lunnon, Hilkka Soininen, Iwona Kloszewska, Magda Tsolaki, Bruno Vellas, Patrizia Mecocci, Simon Lovestone, Stephen Newhouse, Richard Dobson, for the Alzheimers Disease Neuroimaging Initiative

**Affiliations:** 1 National Institute of Health Research Biomedical Research Centre for Mental Health, South London and Maudsley National Health Service Foundation Trust, London, United Kingdom; 2 King's College London, Institute of Psychiatry, London, United Kingdom; 3 Laboratory of Behavioral Neuroscience, National Institute on Aging, Baltimore, Maryland, United States of America; 4 Proteome Sciences plc, Cobham, Surrey, United Kingdom; 5 School of Neurology, University of Eastern Finland and University Hospital of Kuopio, Kuopio, Finland; 6 Department of Old Age Psychiatry and Psychotic Disorders, Medical University of Lodz, Lodz, Poland; 7 3rd Department of Neurology, “G. Papanicolaou” Hospital, Aristotle University of Thessaloniki, Thessaloniki, Greece; 8 Department of Geriatric Medicine, Grontople de Toulouse, Toulouse University Hospital, Toulouse, France; 9 Institute of Gerontology and Geriatrics, University of Perugia, Perugia, Italy; Mental Health Research Institute of Victoria, Australia

## Abstract

Changes in brain amyloid burden have been shown to relate to Alzheimer's disease pathology, and are believed to precede the development of cognitive decline. There is thus a need for inexpensive and non-invasive screening methods that are able to accurately estimate brain amyloid burden as a marker of Alzheimer's disease. One potential method would involve using demographic information and measurements on plasma samples to establish biomarkers of brain amyloid burden; in this study data from the Alzheimer's Disease Neuroimaging Initiative was used to explore this possibility. Sixteen of the analytes on the Rules Based Medicine Human Discovery Multi-Analyte Profile 1.0 panel were found to associate with [^11^C]-PiB PET measurements. Some of these markers of brain amyloid burden were also found to associate with other AD related phenotypes. Thirteen of these markers of brain amyloid burden – c-peptide, fibrinogen, alpha-1-antitrypsin, pancreatic polypeptide, complement C3, vitronectin, cortisol, AXL receptor kinase, interleukin-3, interleukin-13, matrix metalloproteinase-9 total, apolipoprotein E and immunoglobulin E – were used along with co-variates in multiple linear regression, and were shown by cross-validation to explain >30% of the variance of brain amyloid burden. When a threshold was used to classify subjects as PiB positive, the regression model was found to predict actual PiB positive individuals with a sensitivity of 0.918 and a specificity of 0.545. The number of *APOE* ϵ 4 alleles and plasma apolipoprotein E level were found to contribute most to this model, and the relationship between these variables and brain amyloid burden was explored.

## Introduction

The failure of several clinical trials targeting brain amyloid deposition in patients with Alzheimers disease (AD) has led to the suggestion that these agents may be useful if targeted at older individuals in pre-symptomatic stages of the disease [1], [2]. Screening methods that accurately identify at-risk non-demented older individuals who are most likely to benefit from such treatments will therefore represent a major advance in our ability to effectively test these disease-modifying treatments [3]. If clinical trials of amyloid lowering interventions were successful in the pre-symptomatic stages of AD, then there would be a desire to identify non-demented elderly individuals with elevated brain amyloid burden who could potentially benefit from early intervention. However identifying suitable individuals poses a great challenge in terms of feasibility and cost. To date, the two methods that are most likely to be useful in estimating levels of brain amyloid burden are in vivo imaging with positron emission tomography (PET) using radioligands binding to fibrillar amyloid beta (A*β*), such as [^11^C] Pittsburgh B compound (PiB), and assays of A*β* levels in cerebrospinal fluid (CSF) [4], [5]. However, both these methods have inherent drawbacks that limit their utility as screening tools, especially in resource-poor settings. While PET scanning is expensive and limited to specialised centres, lumbar puncture to obtain CSF is associated with some patient discomfort and is unlikely to be used in primary health care centres to routinely screen large numbers of elderly patients. An inexpensive, non-invasive screening method that accurately estimates brain amyloid burden would therefore fulfil a critical unmet need in the care of the elderly.

The identification of blood-based biomarkers associated with AD diagnosis [6]–[9] or distinct endophenotypes of AD pathology such as brain atrophy [10]–[12], hippocampal metabolite abnormalities [13] and amyloid burden [14], have previously been reported. In these studies, proteomic analyses were combined with neuroimaging methods to identify plasma signals associated with measures of AD pathology. In this study, a different strategy was used by examining the association between brain amyloid burden and a panel of 146 plasma analytes – proteins, complexes and metabolites – measured by Rules Based Medicine, Inc. (RBM) (Austin, TX) using the Human Discovery Multi-Analyte Profile (MAP) 1.0 panel and a Luminex 100 platform. Some of the analytes on this panel, such as apolipoprotein E (APOE) and complement C3 have previously been shown to associate with brain amyloid burden [14], [15], while others are associated with other diseases. These assays were performed in plasma samples that were collected from participants in the Alzheimers Disease Neuroimaging Initiative (ADNI; http://adni.loni.ucle.edu) study who also underwent [^11^C]-PiB PET imaging for quantification of fibrillar brain amyloid burden. The main aim of this study was to ask whether concentrations of a panel of plasma proteins and metabolites might accurately reflect the extent of fibrillar amyloid in the brain. A secondary aim was to understand the relationship between the number of *APOE* ϵ 4 alleles and plasma based markers of brain amyloid burden.

## Results

### RBM analytes associate with A*β* levels in the brain

Levels of analytes measured by the RBM Human Discovery MAP 1.0 from ADNI plasma samples were compared to fibrillar amyloid in the RBM-PiB PET cohort (N = 71). Characteristics of this subcohort are summarised in Table 1 where it can be seen that brain amyloid burden was almost significantly different at the 0.05 level between diagnostic groups (Kruskal-Wallis (KW) *χ* ^2^ test p-value 0.055). The distribution of brain amyloid burden in the RBM-PiB PET cohort is shown in Figure S1. In the slightly larger ADNI-PiB PET cohort (i.e. all ADNI subjects with [^11^C] PiB-PET scans performed at baseline), whose sample characteristics are shown in Table S1, brain amyloid burden was found to be significantly different across diagnostic groups (KW p-value 0.022).

**Table 1:** Characteristics of the ADNI RBM-PiB PET cohort.

	Diagnostic group (number of subjects)			
Characteristics	Control (3)	MCI (52)	AD (16)	P-value
Subject age in years at time of plasma sample	77.4 [5.6]	75.4 [11.1]	72.3 [8.2]	0.290
(Median [IQR])				
Sex (Male/Female)	1/2	37/15	10/6	0.263
Years of education (Median [IQR])	13.0 [3.0]	16.0 [5.0]	16.0 [5.3]	0.398
Number of *APOE* ϵ 4 alleles (0/1/2)	2/1/0	25/22/5	7/7/2	0.977
Days between [^11^C]-PiB PET scan and plasma sample (Median [IQR])	5.0 [15.5]	23.5 [60.5]	21.5 [42.3]	0.288
Average PiB uptake	1.31 [0.108]	1.98 [0.723]	1.90 [0.438]	0.055
(Median [IQR])				

Characteristics of the ADNI RBM-PiB PET subcohort by diagnostic group. P-values were calculated for differences across diagnostic groups using a Kruskal-Wallis *χ* ^2^ test for continuous characteristics and simulated contingency p-values for categorical characteristics.

The analytes most associated with brain amyloid burden in the RBM-PiB PET cohort, after taking into account co-variates (age, gender, years of education, number of *APOE* ϵ 4 alleles and the number of days between [^11^C]-PiB PET scan and plasma sample), are shown in Table 2.

**Table 2:** RBM analytes associated with brain amyloid burden.

RBM analyte	Gene name	Uniprot ID	Partial SRC with A*β*	P-value	Benjamini-Hochberg corrected p-value
C-peptide	*INS*	P01308	−0.310	0.010	0.351
Fibrinogen (*α*, *β* and γ)	*FG(A/B/G)*	P02671 P02675 P02679	−0.307	0.010	0.351
Alpha-1-antitrypsin	*SERPINA1*	P01009	−0.302	0.012	0.351
Pancreatic polypeptide	*PPY*	P01298	−0.296	0.014	0.351
Complement C3	*C3*	P01024	−0.296	0.014	0.351
Vitronectin	*VTN*	P04004	−0.295	0.014	0.351
von Willebrand factor	*VWF*	P04275	−0.287	0.017	0.363
Cortisol	(NA)	(NA)	0.271	0.025	0.412
Serum amyloid p-component	*APCS*	P02743	−0.268	0.027	0.412
AXL receptor tyrosine kinase	*AXL*	P30530	0.266	0.028	0.412
Interleukin-3	*IL3*	P08700	0.261	0.032	0.412
Interleukin-13	*IL13*	P35225	0.252	0.038	0.412
Matrix metalloproteinase-9 total	*MMP9*	P14780	−0.250	0.040	0.412
APOE	*APOE*	P02649	−0.248	0.042	0.412
Leptin	*LEP*	P41159	−0.248	0.042	0.412
Immunoglobulin E (IgE)	(NA)	(NA)	−0.243	0.046	0.424

Analytes with a partial SRC p-value of <0.05 are shown. Benjamini-Hochberg corrected p-values were calculated to take into account the comparisons against all RBM analytes.

### Prediction of brain amyloid burden using plasma RBM analytes

To determine if a subset of the RBM panel was able to predict fibrillar amyloid levels in the brain, multiple linear regression was used. In this analysis the following subject co-variates were included: age at plasma sample, years of education, gender, the number of *APOE* ϵ 4 alleles and the difference, in days, between plasma sampling and [^11^C]-PiB PET scan date. Multiple linear regression was applied to predict brain amyloid burden using these co-variates only, giving a leave one out (LOO) cross validation (CV) R^2^ of 0.040. When brain amyloid burden was regressed to *just* the number of *APOE* ϵ 4 alleles this gave a LOO CV R^2^ of 0.123. Then multiple linear regression was applied to predict brain amyloid burden from both RBM analytes and co-variates; the analysis was restricted to the 16 RBM analytes that had a partial Spearmans rank correlation (SRC) uncorrected p-value <0.05 (Table 2) resulting in a LOO CV R^2^ of 0.276.

**Figure 1:**
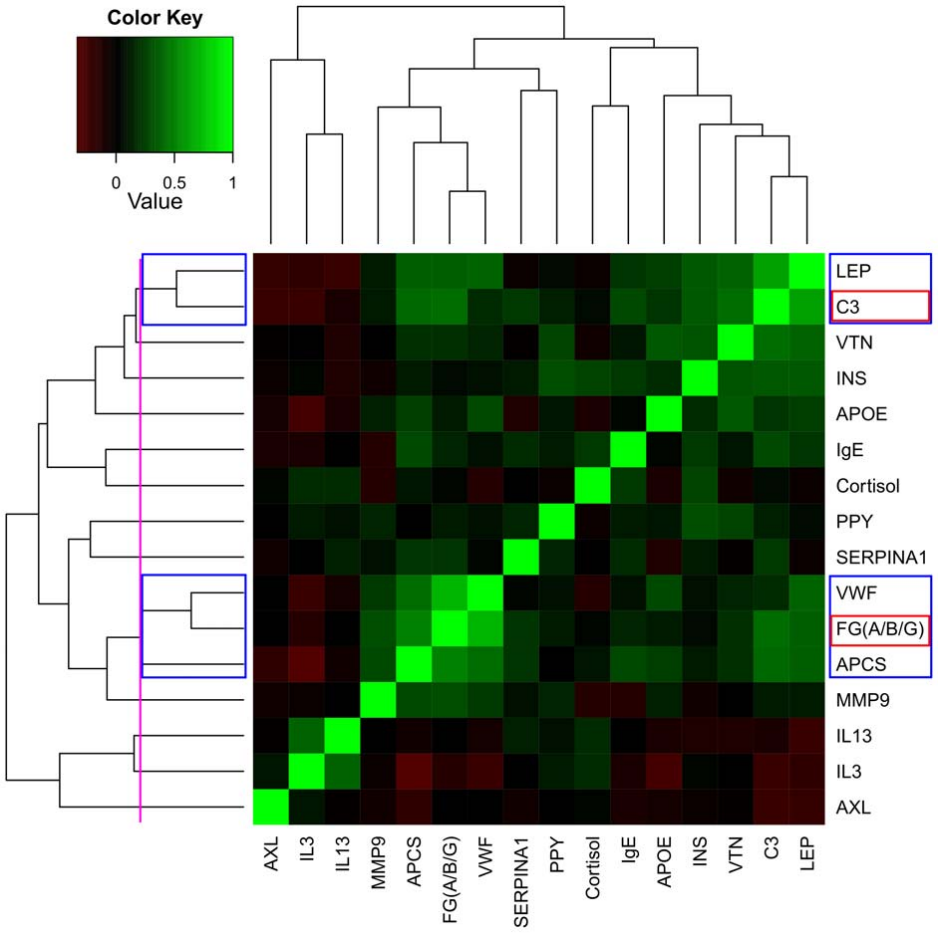
Analyte correlations. Heatmaps of partial SRC between RBM analytes significantly associated (p-value <0.05) with brain amyloid burden, taking into account: age, gender, years of education, number of *APOE* ϵ 4 alleles and the difference, in days, between plasma sampling and [^11^C]-PiB PET scan date. RBM analytes have been ordered by hierarchical clustering, the final cut-off is shown in purple. Variables that have been grouped are shown in blue boxes, and the representative for each group is shown in a red box.

Overfitting was reduced by grouping correlated variables and selecting one RBM analyte to represent each group (Figure 1); first all the 16 RBM analytes were used, then analytes were removed from the model one by one, in the order determined by clustering, and LOO CV repeated. The order of analyte removal was: (1) von willebrand factor, (2) leptin, (3) serum amyloid p-component, (4) vitronectin, (5) interleukin-13, (6) component C3, (7) matrix metalloproteinase-9 total, (8) immunoglobulin E (IgE), (9) APOE, (10) pancreatic polypeptide, (11) alpha-1-antitrypsin, (12) interleukin-3, (13) cortisol, (14) fibrinogen and finally (15) AXL receptor tyrosine kinase.

**Figure 2:**
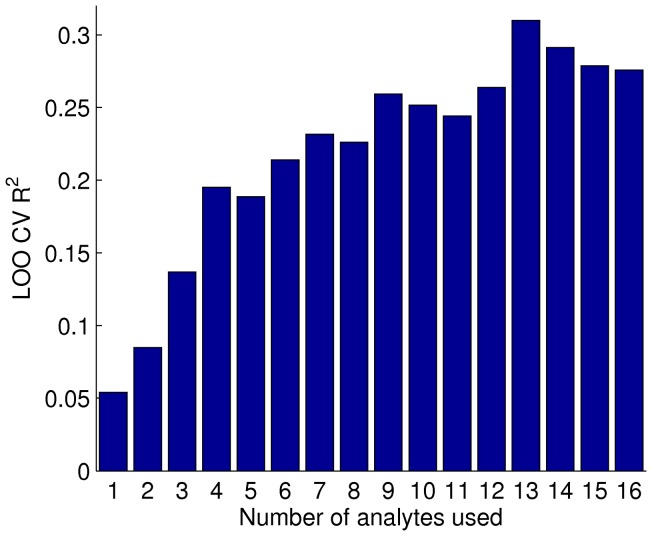
Cross validation of multiple linear regression. Barplot of the LOO CV R^2^ of multiple linear regressions of brain amyloid burden against a range of RBM analytes and co-variates. Subsets of RBM analytes that associated with brain amyloid levels with a p-value <0.05 were used. Various subsets were chosen by hierarchical clustering at various cutoffs, with the analyte most associated with brain amyloid burden in each cluster chosen to represent that cluster. Age, gender, years of education, the number of *APOE* ϵ 4 alleles and the difference, in days, between plasma sampling and [^11^C]-PiB PET scan were used as co-variates.

**Figure 3:**
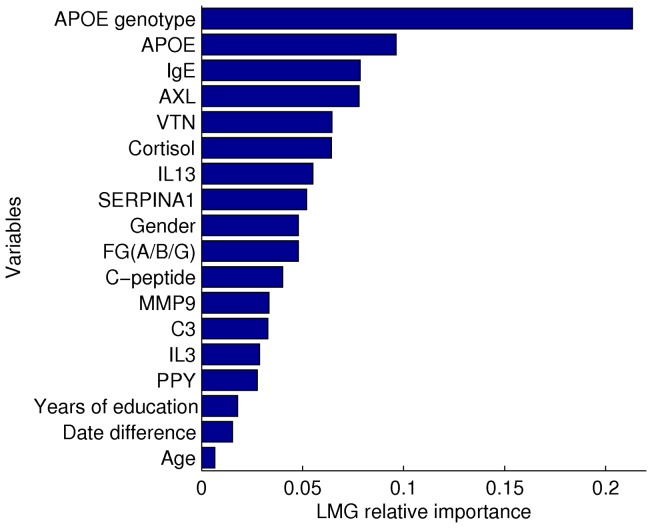
Relative importance in multiple linear regression. Relative importance of variables used in multiple linear regression assessed using the LMG relative importance score [51]. When all the data is used to fit the model it explains 62.0% of the variance of the brain amyloid burden, here the contribution of the variables used in this model are shown.

The grouping that resulted in the highest LOO CV R^2^ over all possible hierarchical clustering cut-offs was used (Figure 2). This was achieved when 13 RBM analytes were used: c-peptide, fibrinogen, alpha-1-antitrypsin, pancreatic polypeptide, complement C3, vitronectin, cortisol, AXL receptor tyrosine kinase, interleukin-3, interleukin-13, matrix metalloproteinase-9 total, APOE and IgE (LOO CV R^2^ 0.310, permutation test p-value 4×10^−5^). The 13 RBM analyte and co-variate model is able to account for approximately a third of the variance of brain amyloid burden. The relative importance of variables to the model is shown in Figure 3; the number of *APOE* ϵ 4 alleles was seen to be the most important variable for the model, but RBM analytes contribute more to the model than years of education or age. The 71 subjects brain amyloid burden was then dichotomised into either PiB positive (brain amyloid burden >1.5) or PiB negative (brain amyloid burden <1.5), based on a threshold used in relevant literature [16]. Similarly, the subjects LOO CV predicted brain amyloid burden, based on the 13 RBM and co-variate model, was dichotomised into either predicted PiB positive (predicted brain amyloid burden >1.5) or predicted PiB negative (predicted brain amyloid burden <1.5). For each subject the predicted and actual PiB classes (positive or negative) were compared; the predicted PiB classes identified actual PiB positive subjects with a sensitivity of 0.918 and a specificity of 0.545. It was also found that removing all co-variates, except the number of *APOE* ϵ 4 alleles, from the 13 RBM and co-variate regression model gave a similar but slightly improved predictive ability (LOO CV R^2^ 0.311).

### Markers of brain amyloid burden associate with other measures of AD pathology

The 16 RBM analytes found to associate with brain amyloid burden in the ADNI RBM-PiB PET cohort were then tested for association with other phenotypes known to relate to AD pathology in the ADNI-RBM cohort (sample characteristics shown in Table S2). In this analysis age, gender, years of education and the number of *APOE* ϵ 4 alleles were included in partial correlation analysis. Subjects with missing values for a relevant comparison were excluded. First the level of the markers of brain amyloid burden were compared with the level of A*β*_1−42_ in the CSF. Leptin was found to associate with CSF A*β*_1−42_ (partial SRC 0.183, BH MTC p-value 0.0183). Additionally, when multiple testing was not taken into account, vitronectin was associated with CSF A*β*_1−42_ (partial SRC 0.131, uncorrected p-value 2.07×10^−2^).

Markers of brain amyloid burden were then compared to AD relevant brain regions as measured by structural magentic resonance imaging (sMRI). The volume of the left and right hippocampi, and the thickness of the left and right entorhinal cortices were used, as these regions are known to be important in AD pathology [12]. Alpha-1-antitrypsin was found to associate at the 5% significance level with the thickness of both the left (partial SRC −0.132, BH MTC p-value 0.0289) and right (partial SRC −0.145, BH MTC p-value 9.82×10^−3^) entorhinal cortices. Additionally, leptin was found to associate with the thickness of the right entorhinal cortex (partial SRC 0.124, BH MTC p-value 0.0264). Cortisol was found to associate at the 5% significance level with the volume of both the left (partial SRC −0.158, BH MTC p-value 2.71×10^−3^) and right hippocampi (partial SRC −0.161, BH MTC p-value 2.07×10^−3^).

Different cognitive tests, such as the Mini Mental State Exam (MMSE) and Alzheimer's Disease Assessment Scale-cognitive subscale (ADAS-cog) 13, assess different aspects of cognitive decline. The association between scores from these tests and levels of markers of brain amyloid burden was analysed; 4/16 of the markers of brain amyloid burden – alpha-1-antitrypsin (partial SRC −0.145, BH MTC p-value 3.02×10^−3^), complement C3 (partial SRC −0.189, BH MTC p-value 9.46×10^−5^), cortisol (partial SRC −0.162, BH MTC p-value 8.33×10^−4^) and fibrinogen (partial SRC −0.130, BH MTC p-value 8.28×10^−3^) – were found to associate with the total MMSE score at the 5% level. In the same cohort (ADNI-RBM), three of these markers – alpha-1-antitrypsin (partial SRC 0.172, BH MTC p-value 0.0403), complement C3 (partial SRC 0.119, BH MTC p-value 0.0467) and fibrinogen (partial SRC 0.111, BH MTC p-value 6.34×10^−4^) – were found to associate with the ADAS-cog 13 score at the 5% significance level.

Levels of the 16 markers of brain amyloid burden were then compared between different diagnostic groups (control, MCI and AD) in the various cohorts, to assess whether these markers were related to clinical diagnosis. It was found that half of the biomarkers of brain amyloid burden measured – APOE (BH MTC KW p-value 1.24×10^−9^, AD/control median difference (MD) −0.143), complement C3 (BH MTC KW p-value 8.88×10^−8^, MCI/control MD −0.0483), cortisol (BH MTC KW p-value 2.40×10^−3^, AD/control MD 0.0366), interleukin-3 (BH MTC KW p-value 8.48×10^−3^, AD/control MD −0.0670), leptin (BH MTC KW p-value 8.48×10^−3^, AD/control MD −0.112), pancreatic polypeptide (BH MTC KW p-value 1.92×10^−2^, AD/control MD 0.122), alpha-1-antitrypsin (BH MTC KW p-value 1.75×10^−7^, AD/control MD 0.0300) and vitronectin (BH MTC KW p-value 5.50×10^−3^, AD/control MD -0.0344) – significantly differ at the 5% level between diagnostic groups.

### The effect of *APOE* genotype on APOE level in plasma and brain amyloid burden

The number of *APOE* ϵ 4 alleles and the level of APOE in plasma were the two variables that contributed most to the regression model. A number of studies have shown that APOE level in plasma is affected by presence of *APOE* ϵ 4 alleles [14], [15], [17]–[19]. Additionally, Slooter et al., [17] have previously shown that the difference in plasma APOE levels between AD and control subjects is largely driven by the *APOE* genotype, and so the interaction of these two variables was studied further. In the ADNI-RBM cohort the number of *APOE* ϵ 4 alleles was seen to have a negative effect on plasma APOE levels (KW p-value <2.20×10^−16^, Table 3). The negative effect of *APOE* genotype on plasma APOE levels in this ADNI subcohort was demonstrated recently using Analysis of Variance (ANOVA) [20]. The analysis presented here shows that this result holds when assumptions of normality are dropped. The negative effect of the number of *APOE* ϵ 4 alleles on plasma APOE levels observed fits with the findings of some literature [15], [17], [18], but is the opposite of the positive effect seen by both Evans et al. and Thambisetty et al. (2010) [14], [19].

**Table 3:** The effect of *APOE* genotype on plasma APOE levels.

	The plasma level in log μg/ml of APOE in			
	subjects with n *APOE* ϵ 4 alleles (median [IQR])			
Cohort	n = 0	n = 1	n = 2	Kruskal-Wallis χ^2^ P-value
ADNI-RBM	1.79 [0.200]	1.66 [0.203]	1.52 [0.233]	<2.20×10^−16^
ANM + KHPDCR	1.91 [9.75×10^−2^]	1.88 [9.00×10^−2^]	1.82 [7.00×10^−2^]	<2.20×10^−16^
ANM + KHPDCR				
controls	1.9 [0.100]	1.88 [8.00×10^−2^]	1.83 [3.00×10^−2^]	1.57×10^−4^

Level of plasma APOE stratified by the subjects number of *APOE* ϵ 4 alleles.

Given the discrepancy between this finding and those in two published studies, the relationship between the number of *APOE* ϵ 4 alleles and plasma APOE levels was studied in an independent cohort of 694 subjects (AddNeuroMed and King's Health Partners Dementia Case Register, ANM + KNPDCR). In the ANM + KHPDCR cohort the number of *APOE* ϵ 4 alleles was also seen to have a negative effect on plasma APOE levels (KW p-value <2.20×10^−16^, Table 3). Both studies that have found a positive relationship between *APOE* ϵ 4 alleles on plasma APOE levels have been conducted in cohorts of cognitively normal subjects [14], [19], which may account for this inconsistency. However, a similar negative effect was found in the control subjects (who are cognitively normal) of the ANM + KHPDCR cohort (KW p-value 1.57×10^−4^, Table 3). This suggests another factor, other than cognitive decline, is responsible for the discrepancies between these studies.

Given that the number of *APOE* ϵ 4 alleles affects both plasma APOE levels and brain amyloid burden, it is possible that the number of *APOE* ϵ 4 alleles confounds the association between plasma APOE levels and brain amyloid burden. To test this, partial correlation analysis was repeated excluding the number of *APOE* ϵ 4 alleles, this increased the correlation of plasma APOE level and brain amyloid burden (partial SRC −0.393, p-value 6.9×10^−4^), which indicated that the association is indeed partly confounded by the number of *APOE* ϵ 4 alleles.

## Discussion

In this study, fibrillar amyloid beta levels, in ADNI subjects, have been compared and related to the level of analytes on the RBM panel in plasma. Brain amyloid burden appears to be distributed bimodally in the RBM-PiB PET cohort, as has been previously reported for a larger ADNI subcohort by Ewers et al. [21]. Associations of C-peptide, fibrinogen, alpha-1-antitrypsin, pancreatic polypeptide, complement C3, vitronectin, von willebrand factor, cortisol, serum amyloid p-component, AXL receptor tyrosine kinase, interleukin-3, interleukin-13, matrix metallproteinase-9, APOE, leptin and immunoglobulin E with brain amyloid burden have been found in this study. Some of these markers of brain amyloid burden were also found to associate with other AD related phenotypes, such as CSF A*β*_1−42_, MRI features, cognitive tests and diagnostic groups. In regression models it was found that models including both RBM analytes and co-variates performed better than those using only co-variate information, suggesting that the RBM panel of analytes can be used as markers of brain amyloid burden. Combining highly correlated variables was found to reduce overfitting and led to a set of 13 RBM analytes that together with co-variates could explain >30% of the variance of brain amyloid burden and predict PiB positive individuals with a high sensitivity. This result, and the increased predictive accuracy of the 13 RBM model in comparison to using only co-variates to predict fibrillar amyloid alone, indicate that these analytes reflect levels of fibrillar amyloid in the brain.

The potential of APOE level in plasma to be used as a biomarker for brain amyloid burden, previously shown in Thambisetty et al. (2010) [14], is given support by this study. However it should be noted that the association between plasma APOE level and brain amyloid burden was seen to be positive in that study and negative in this. This inconsistency may relate to differences between the cohorts, for example the BLSA cohort studied in Thambisetty et al. (2010) were selected to be cognitively normal. This fits with the recent finding that plasma level of APOE correlates negatively with brain amyloid burden in the Australian Imaging, Biomarker and Lifestyle Flagship Study of Ageing, which includes subjects suffering from AD [15]. Similarly, the effect of the presence of *APOE* ϵ 4 alleles on plasma APOE level in the study presented here was found to be the opposite of that found in both Thambisetty et al. (2010) and Evans et al. [14], [19], however it is the same as that seen in Slooter et al., Siest et al. and Gupta et al. [15], [17], [18]. Additionally, this relation was replicated in an independent cohort (ANM + KHPDCR) and it's control subcohort. This latter finding suggests that the differences between the findings of these studies is not due to the subjects cognitive status. The factor/s responsible for these inconsistencies are not known, but the fact that strong associations are seen in all studies is encouraging.

While no association between the level of RBM analytes in plasma and brain amyloid burden was found to be significant at the 5% level after multiple testing corrections, it should be noted the cohort used (RBM-PiB PET) contained relatively few subjects and that many analytes that associated significantly at the uncorrected 0.05 level have known relations to AD pathology, for example levels of APOE and complement C3 in plasma have previously been found to associate with fibrillar amyloid levels in the Baltimore Longitudinal Study of Aging (BLSA) cohort [14]. In addition, some of these analytes were subsequently found to associate with surrogate phenotypes of AD pathology in the larger ADNI-RBM cohort, such as: diagnostic groups, MMSE score, ADAS-cog 13 score, CSF A*β*_1−42_ level, and the thickness and/or volume of the entorhinal cortices. However, 7/16 of the markers of brain amyloid burden – c-peptide, von Willebrand factor, serum amyloid p-component, AXL receptor tyrosine kinase, interleukin-13, matrix metalloproteinase-9 total and IgE – were not found to associate with any of the surrogate phenotypes of AD pathology that were tested. It should be noted that 5 of the 7 are linked to AD in the literature (Table S3), only IgE and interleukin-13 have no prior reported association. Most of these surrogate phenotypes, except CSF A*β*_1−42_, are believed to change at a later disease stage than amyloid pathology, and so it is possible that the lack of association of some of the markers with, for example, diagnostic groups is due to the mixture of high and low brain amyloid burden control subjects used. However, the lack of association of the majority of the markers with CSF A*β*_1−42_ levels is of greater concern because CSF A*β*_1−42_ levels are strongly associated with brain amyloid burden [4], [5]; this may indicate that we are over-fitting the available data and further highlights the need for datasets with larger sample sizes for future studies of markers of brain amyloid burden.

While half of the markers associated with diagnostic groups, 3/8 of these markers – alpha-1-antitrypsin, pancreatic polypeptide and interleukin-3 – had a median difference between AD (or MCI) and control subjects that was of the opposite sign to the partial SRC coefficient measuring their association with brain amyloid burden. This result is surprising as brain amyloid burden is positively associated with AD and MCI diagnosis groups. This discrepancy could relate to the delay between these disease stages, and may mean that the level of some of the markers in plasma changes during A*β* deposition and then changes again, but in the opposite direction, before the onset of clinical symptoms. Similar u-shaped profiles, but between subjects in different diagnostic groups, have been observed (cross-sectionally) in the level of many leukocyte transcripts during AD progression [22].

Partial correlation showed that the number of *APOE* ϵ 4 alleles partly confounded the association between APOE level in plasma and brain amyloid burden; however, plasma APOE levels did help a regression model predict brain amyloid burden and so further study is required to get a clearer idea of the *APOE* ϵ 4 independent information conveyed by plasma APOE levels. This study has revealed many novel potential markers of brain amyloid burden, chosen to give *APOE* ϵ 4 independent information, as well as replicating findings from other studies. This will allow further validation work that can test the replicability and clinical utility of these markers.

In a previous study that used discovery proteomics to identify proteins associated with brain amyloid levels, Thambisetty et al. (2010) showed that levels of APOE and Complement C3 precursor in plasma were different between subjects with high and low brain amyloid burdens [14]. It was encouraging that both were seen to be associated with brain amyloid burden in this study as well. Complement C3 precursor has also been found to be associated with atrophy of hippocampal volume, another imaging marker of AD [11], and to have a role in plaque clearance in a mouse model [23]. It has also been found along with vitronectin to be at different levels in serum between control and AD subjects [6]. The level of fibrinogen gamma was also found to be associated with atrophy of hippocampal volume in Thambisetty et al. (2011) [11]. Fibrinogen alpha, beta and gamma are targeted by the same RBM analyte and were found to associate with brain amyloid burden in this study.

Of the 16 RBM analytes whose level in plasma associated with brain amyloid burden, many have known relationships with Alzheimer's disease. The levels of the following have previously been found to be different between control and AD subjects: alpha-1-antitrypsin [9], [24], APOE [9], cortisol [9], [25], [26], interleukin-3 [27], matrix metalloproteinase-9 [9], [28], pancreatic polypeptide [9], [29], serum amyloid p-component [30] and von Willebrand factor [31]. Serum amyloid p-component [32] and insulin [33] have been shown to affect ‘AD’-like pathology *in vitro*. Interleukin-3 [34] and leptin [35] have been found to affect the interaction of neurons and A*β*. Additionally, interleukin-13 has been found to be produced in microglia in response to A*β* [36]. More recently, APOE and matrix metalloproteinase-9 have been shown to be involved together in the breakdown of the blood brain barrier, which can initiate neurodegeneration [37].

Given the relatively small number of subjects in this study it was not practical to separate the subjects into training and test sets, to assess the predictive accuracy of the regression model. Instead, k-fold cross-validation was used, allowing more of the subjects to be used for fitting the regression model. Generally it is advisable to use 10-fold cross-validation because it has been found to have a lower variance [38]. However, given the limited number of samples available, a leave one out cross-validation approach was used in this study to allow the maximal use of the subjects available. Given the limited number of subjects on which the model is based, it will be important in the future to study the ability of these biomarkers to predict brain amyloid burden in an independent cohort. Validation studies would benefit from greater numbers of subjects and better sampling strategies. For example, the distribution of brain amyloid burden in the RBM-PiB PET subcohort is affected by the sampling strategy applied to select control subjects for RBM measurements; only plasma of control subjects with high CSF A*β*_1−42_ were selected, for reasons unrelated to the current study. This means that the resulting model may not extrapolate to cognitively normal subjects with high brain amyloid burden, this could be tested in a validation study. Additionally, the use of only three control subjects may make the regression model less likely to generalise to prediction of brain amyloid burden in early Alzheimer's disease.

In conclusion, analytes associating with brain amyloid burden have the potential to act as biomarkers of early AD-related pathology. In this study sixteen analytes were found to associate with brain amyloid burden, including two (APOE and complement C3) that had had already been shown to associate with brain amyloid burden in an independent cohort. Some of these analytes were also found to associate with other AD related phenotypes in a larger ADNI subcohort, such as: CSF A*β*_1−42_, MRI features, cognitive scores and diagnostic groups. Some of these analytes were found to correlate highly with each other, and so a representative set of thirteen analytes – c-peptide, fibrinogen, alpha-1-antitrypsin, pancreatic polypeptide, complement C3, vitronectin, cortisol, AXL receptor kinase, interleukin-3, interleukin-13, matrix metalloproteinase-9 total, APOE and IgE – were used along with subject age, gender, years of education, the number of *APOE* ϵ 4 alleles and sampling dates to predict brain amyloid burden. The 13 analyte and co-variate model was found by cross-validation to account for >30% of the variance of brain amyloid burden, as opposed to ∼ 4–13% using just co-variates alone, showing the potential of plasma analytes as markers of brain amyloid burden. The model was also able to predict PiB positive individuals with a high sensitivity. The two variables with the largest contribution to the model were found to be the number of *APOE* ϵ 4 alleles and plasma APOE level. The association of plasma APOE level with brain amyloid burden was shown to be partly confounded by the number of *APOE* ϵ 4 alleles, highlighting the importance of novel biomarkers that are less confounded by the *APOE* genotype revealed by this study.

## Materials and Methods

### Ethics statement

Written informed consent was obtained from all participants in ADNI and the study was conducted with prior institutional ethics approval. Both ANM and KHPDCR were approved by the South London and Maudsley NHS Foundation Trust ethics committee. Ethics committee approval was also obtained at each of the participating centres in accordance with the Alzheimers Associations published recommendations [39].

### ADNI Data

Data used in the preparation of this article were obtained from the ADNI database (adni.loni.ucla.edu). The ADNI was launched in 2003 by the National Institute on Aging (NIA), the National Institute of Biomedical Imaging and Bioengineering (NIBIB), the Food and Drug Administration (FDA), private pharmaceutical companies and non-profit organisations, as a $60 million, 5- year public-private partnership. The primary goal of ADNI has been to test whether serial magnetic resonance imaging (MRI), positron emission tomography (PET), other biological markers, and clinical and neuropsychological assessment can be combined to measure the progression of mild cognitive impairment (MCI) and early Alzheimers disease (AD). Determination of sensitive and specific markers of very early AD progression is intended to aid researchers and clinicians to develop new treatments and monitor their effectiveness, as well as lessen the time and cost of clinical trials.

The Principal Investigator of this initiative is Michael W. Weiner, MD, VA Medical Center and University of California San Francisco. ADNI is the result of efforts of many co-investigators from a broad range of academic institutions and private corporations, and subjects have been recruited from over 50 sites across the U.S. and Canada. The initial goal of ADNI was to recruit 800 adults, ages 55 to 90, to participate in the research, approximately 200 cognitively normal older individuals to be followed for 3 years, 400 people with MCI to be followed for 3 years and 200 people with early AD to be followed for 2 years. For up-to-date information, see www.adni-info.org.

Demographic (age, gender, years of education), genetic (number of *APOE* ϵ 4 alleles), diagnosis (control, MCI or AD at a given date) and analyte (metabolite/protein/complex) levels in plasma were compared with brain amyloid burden (or other markers of AD pathology such as: CSF A*β*_1−42_ level, MRI features, cognitive scores or diagnostic groups). Diagnoses were recorded for each subject at each visit. Plasma and CSF were collected from fasted subjects using the procedures described previously [29], [40]. Levels of 190 analytes were measured from subject plasma using the Rules Based Medicine (RBM, rulesbasedmedicine.com, Austin, TX) Human Discovery Multi-Analyte Profile (MAP) 1.0 panel and a Luminex 100 platform [41]. Measurements of 44 analytes were excluded on the basis of quality control, leaving 146 analytes in the subsequent analysis. The levels of all 146 analytes (except: apolipoprotein H, complement factor H, E selectin, epidermal growth factor, fibrinogen, interleukin-12 subunit p40, placenta growth factor, serum glutamic oxaloacetic transaminase and thrombopoietin) were log transformed to improve the fit of the levels to the normal distribution. Description of methods used to derive measurements of regional [^11^C] PiB-PET levels have been given in Jagust et al. (2010) and (2011) previously [16], [42]. In this study a similar approach was taken by averaging over regional [^11^C] PiB-PET measurements in parietal, frontal, anterior cingulate and precuneus regions of interest, to derive a global measure of brain amyloid burden.

Data from either baseline or 12 months were used for these cohorts, as described above, chosen to increase the number of subjects with available data. Eighty four subjects (ADNI-PiB PET cohort) had a [^11^C] PiB-PET scan 12 months after baseline, the characteristics of this sample are shown in Table S1. Five hundred and sixty six subjects (ADNI-RBM cohort) had RBM analytes measured in plasma collected at baseline, the characteristics of this sample at baseline is shown in Table S2. Seventy one subjects (RBM-PiB PET cohort) had RBM analytes measured in plasma collected 12 months after baseline and a [^11^C] PiB-PET scan within a year of this, the characteristics of this sample at the date of plasma collection is shown in Table 1. Converters from control to MCI between plasma sample and [^11^C] PiB-PET scan date account for the discrepancy in diagnostic groups between the ADNI-PiB PET and RBM-PiB PET cohorts. Subject age was determined based on the same dates.

Sample characteristics of each cohort by diagnostic group were analysed in R [43]. Many continuous variables were not distributed normally, and so are described by median and interquartile range instead of mean and standard deviation, this was also the reason that non-parametric statistical tests were used. Continuous characteristics were tested for differences over diagnostic groups by the non-parametric Kruskal-Wallis *χ* ^2^ test, using kruskal.test in the R stats package. Discrete characteristics were tested for differences over diagnostic groups by simulated contingency table p-values, in the fisher.test function in the R stats package, using 2,000 Monte Carlo samples.

### MRI scan analysis

Dicom format MRI data was downloaded from the ADNI website (www.loni.ucla.edu/ADNI). Data from 1.5 T scanners was used with data collected from a variety of MR-systems with protocols optimised for each type of scanner. The MRI protocol included a high resolution sagittal 3D T1-weighted MPRAGE volume (voxel size 1.1×1.1×1.2 mm^3^) acquired using a custom pulse sequence specifically designed for the ADNI study to ensure compatibility across scanners. Full brain and skull coverage was required for the MRI datasets and detailed quality control carried out on all MR images according to previously published quality control criteria [44], [45].

We applied the Freesurfer pipeline (version 4.5.0) to the MRI images to produce regional cortical thickness and volumetric measures as previously described [46] to produce hippocampal and entorhinal cortex volumes, as well as entorhinal cortical thickness. All volumetric measures from each subject were normalised by the subjects intracranial volume. Cortical thickness measures were not normalised [47] and were used in their raw form.

### Correlation analysis

R was used to analyse SRC and partial SRC, using cor.test in the stats package and pcor.test in the ppcor package [48] respectively. Partial correlations were used to take into account subjects: age, gender, years of education, number of *APOE* ϵ 4 alleles and the number of days separating the date of [^11^C]-PiB PET scan and plasma sample. In the case of correlations between RBM analytes and CSF A*β*, ADAS-cog 13 or MRI features, subjects whose relevant data was missing were excluded. RBM analytes were clustered based upon (1 – their partial SRC) using the R function hclust in the stats package with default settings, and displayed using function heatmap.2 from the R gplots package [49].

### Linear regression

Linear regression was performed using the lm function in the R stats package. This was appropriate because although many variables were not distributed normally, the residuals of the regression models used were approximately. Before regression, measurements of each RBM analyte were transformed to a standard deviation of one to allow each analyte to have equal influence on the model (but not transformed to a mean of zero, to make the analysis more comparable with that used in Thambisetty et al. (2010) [14]). LOO CV was performed by fitting linear regression models to the data, leaving out one subject at a time, and using the model to predict brain amyloid burden in that subject based on the fitted model. LOO CV R^2^ was calculated as the square of the Pearson's correlation coefficient, calculated using cor.test, between the predicted and observed brain amyloid burden.

A cut-off of 1.5 was used to dichotomise brain amyloid burden in the RBM-PiB PET cohort as PiB positive (49 subjects) or negative (22 subjects), as previously suggested by Jagust et al. (2010) [16]. Predicted values were similarly dichotomised. Sensitivity and specificity of this prediction was calculated in R using epi.test in the epiR package [50].

Permutation tests were performed by permuting brain amyloid burden across subjects in the data, and re-calculating the LOO CV R^2^ that was achieved by fitting the regression model to the resulting data. 100,000 permutations were used, and the number of cases in which the LOO CV R^2^ exceeded that achieved with the original dataset was recorded. Relative importance was calculated using the LMG score [51], using the R package relaimpo [52].

### Independent cohort data for validation of the effect of *APOE* ϵ 4 genotype on APOE level in plasma

EDTA plasma samples from fasted subjects were obtained from two independent cohorts: ANM a multicentre European study across six centres [53] and KHPDCR a UK based study. The combined cohort contained 269 control, 163 MCI and 262 AD subjects. *APOE* genotype was determined using DNA extracted from blood leukocytes by a standard phenol-chloroform extraction. The three main alleles *APOE* ϵ 2, *APOE* ϵ 3 and *APOE* ϵ 4 differ at two residues, so consist of a two single nucleotide polymorphism (SNP) haplotype. The SNPs rs429358 and rs7412 were genotyped and the allele inferred. SNPs were determined by allelic discrimination assays based on fluorogenic 5′ nuclease activity. TaqMan SNP genotyping assays were performed on an ABI Prism 7900HT and analyzed using SDS software, according to the manufacturer's instructions (Applied Biosystems, Warrington, UK). 199 control, 103 MCI and 120 AD subjects were found to have 0 *APOE* ϵ 4 alleles. 65 control, 56 MCI and 112 AD subjects were found to have 1 *APOE* ϵ 4 alleles. 5 control, 4 MCI and 30 AD subjects were found to have 2 *APOE* ϵ 4 alleles. The Human Neurodegenerative Panel 1 (7-plex) Cat. HNDG1-36K MILLIPLEX MAP multiplex panels, developed by Merck Millipore, was used to measure APOE level in plasma.

## Supporting Information

Figure S1 **Distribution of brain amyloid burden.** A stacked histogram showing the distribution of brain amyloid burden for different diagnostic groups. Control (dark blue), MCI (green) and AD (red) represent subjects who remained in these diagnostic groups throughout follow up period. Control/MCI (light blue) and MCI/AD (orange) represents subjects whose diagnosis converted between these groups during the follow up period. Brain amyloid burden is in relative units.
(TIF)

Table S1 **Characteristics of the ADNI-PiB PET cohort by diagnostic group.** P-values were calculated when appropriate for differences across diagnostic groups, using a Kruskal-Wallis *χ* ^2^ test for continuous characteristics and simulated contingency table p-values for discrete characteristics.
(PDF)

Table S2 **Characteristics of the ADNI-RBM cohort by diagnostic group.** P-values were calculated when appropriate for differences across diagnostic groups, using a Kruskal-Wallis *χ* ^2^ test for continuous characteristics and simulated contingency table p-values for discrete characteristics.
(PDF)

Table S3 **Association of markers with AD phenotypes.**
(PDF)
