# Supplementary figures and images for: Plasma Based Markers of [11C] PiB-PET Brain Amyloid Burden
Source: PLoS One. 2012 Sep 24;7(9):e44260. doi: 10.1371/journal.pone.0044260 (PMC3454385; doi:10.1371/journal.pone.0044260)

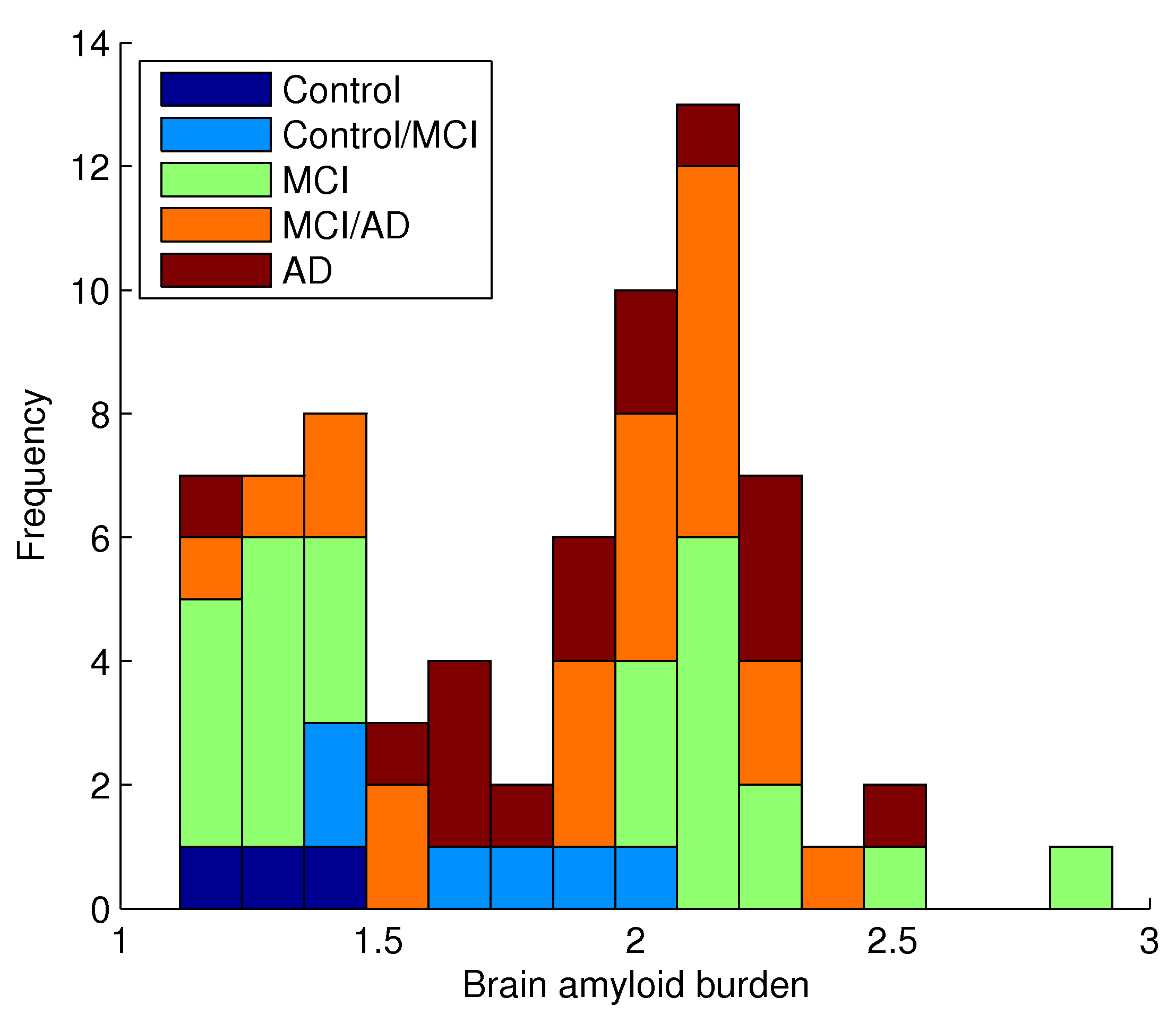

Supplement: Figure S1 — Distribution of brain amyloid burden. A stacked histogram showing the distribution of brain amyloid burden for different diagnostic groups. Control (dark blue), MCI (green) and AD (red) represent subjects who remained in these diagnostic groups throughout follow up period. Control/MCI (light blue) and MCI/AD (orange) represents subjects whose diagnosis converted between these groups during the follow up period. Brain amyloid burden is in relative units. (TIF) [file pone.0044260.s001.tif]
