# Supplementary material for: Plasma Based Markers of [11C] PiB-PET Brain Amyloid Burden
Source: PLoS One. 2012 Sep 24;7(9):e44260. doi: 10.1371/journal.pone.0044260 (PMC3454385; doi:10.1371/journal.pone.0044260)
Supplement: Table S1 — Characteristics of the ADNI-PiB PET cohort by diagnostic group. P-values were calculated when appropriate for differences across diagnostic groups, using a Kruskal-Wallis χ 2 test for continuous characteristics and simulated contingency table p-values for discrete characteristics. (PDF) [file pone.0044260.s002.pdf]

Table S1. Characteristics of the ADNI-PiB PET cohort by diagnostic group.

	Diagnostic group (number of subjects)			
Characteristics	Controls (19)	MCI (49)	AD (16)	P-value
Age in years at time of plasma sample (Median [IQR])	78.9 [10.1]	75.2 [10.4]	72.7 [7.8]	0.222
Sex (Male/Female)	11/8	33/16	10/6	0.782
Years of education (Median [IQR])	14.0 [5.5]	16.0 [4.0]	16.0 [3.8]	0.221
Number of *APOE* ϵ4 alleles (0/1/2)	13/6/0	26/19/4	6/8/2	0.3388
Average PiB uptake (Median [IQR])	1.36 [0.69]	1.98 [0.74]	1.87 [0.408]	0.022

P-values were calculated when appropriate for differences across diagnostic groups, using a Kruskal-Wallis χ^2 test for continuous characteristics and simulated contingency table p-values for discrete characteristics.
