# Supplementary material for: Plasma Based Markers of [11C] PiB-PET Brain Amyloid Burden
Source: PLoS One. 2012 Sep 24;7(9):e44260. doi: 10.1371/journal.pone.0044260 (PMC3454385; doi:10.1371/journal.pone.0044260)
Supplement: Table S2 — Characteristics of the ADNI-RBM cohort by diagnostic group. P-values were calculated when appropriate for differences across diagnostic groups, using a Kruskal-Wallis χ 2 test for continuous characteristics and simulated contingency table p-values for discrete characteristics. (PDF) [file pone.0044260.s003.pdf]

Table S2. Characteristics of the ADNI-RBM cohort by diagnostic group.

		Diagnostic group (number of subjects)			
Characteristics	Summary variables	Control (58)	MCI (396)	AD (112)	P-value
Subject age in years at baseline	Median [IQR]	73.3 [7.88]	75.3 [10.0]	76.0 [11.0]	0.922
	% of missing values	0	7.58 x 10^{-3}	0	1
Sex	Male/Female	30/28	257/139	65/47	0.0915
	% of missing values	0	0	0	NA
Years of education	Median [IQR]	16 [4.75]	16 [4.00]	16 [5.00]	0.268
	% of missing values	0	0	0	NA
Number of APOE ϵ4 alleles	0/1/2	53/5/0	185/164/47	36/53/23	5.00 x 10^{-4}
	% of missing values	0	0	0	NA
MMSE score	Median [IQR]	29 [1.75]	27 [3]	24 [3]	5.65 x 10^{-50}
	% of missing values	0	0	0	NA
ADAS-cog 13	Median [IQR]	9.50 [5.92]	18.3 [8.33]	28.3 [11.4]	5.34 x 10^{-46}
	% of missing values	0	0.758	1.79	NA
Normalised left hippocampal volume	Median [IQR]	2.36 x 10^{-3} [4.30 x 10^{-4}]	1.98 x 10^{-3} [5.08 x 10^{-4}]	1.74 x 10^{-3} [4.29 x 10^{-4}]	4.25 x 10^{-21}
	% of missing values	3.45	1.01	0.893	0.232
Normalised right hippocampal volume	Median [IQR]	2.40 x 10^{-3} [3.45 x 10^{-4}]	2.03 x 10^{-3} [5.09 x 10^{-4}]	1.83 x 10^{-3} [4.04 x 10^{-4}]	7.75 x 10^{-17}
	% of missing values	3.45	1.01	0.893	0.232
Left entorhinal thickness	Median [IQR]	3.19 [0.467]	3.19 [0.661]	2.77 [0.575]	6.55 x 10^{-17}
	% of missing values	3.45	1.01	0.893	0.232
Right entorhinal thickness	Median [IQR]	3.63 [0.508]	3.32 [0.670]	2.98 [0.733]	3.72 x 10^{-15}
	% of missing values	3.45	1.01	0.893	0.232
CSF Aβ_{1-42}	Median [IQR]	247 [28.6]	148 [77.1]	138 [33.4]	9.79 x 10^{-22}
	% of missing values	6.90	56.3	23.2	5.00 x 10^{-4}

P-values were calculated when appropriate for differences across diagnostic groups, using a Kruskal-Wallis χ^2 test for continuous characteristics and simulated contingency table p-values for discrete characteristics.
