# Supplementary material for: Plasma Based Markers of [11C] PiB-PET Brain Amyloid Burden
Source: PLoS One. 2012 Sep 24;7(9):e44260. doi: 10.1371/journal.pone.0044260 (PMC3454385; doi:10.1371/journal.pone.0044260)
Supplement: Table S3 — Association of markers with AD phenotypes. (PDF) [file pone.0044260.s004.pdf]

Table S3. Association of markers with AD phenotypes.

Plasma analyte	Associations in ADNI-RBM	Associations in other cohorts using RBM	Associations in non-RBM studies
C-peptide			'AD like' pathology in vitro [31]
Fibrinogen	MMSE, ADAS-cog13		Hippocampal volume [6]
Alpha-1-antitrypsin	Thickness of the left and right entorhinal cortices, MMSE, ADAS-cog13, diagnostic groups	Diagnostic groups [9]	Diagnostic groups [24]
Pancreatic polypeptide	Diagnostic groups	Diagnostic groups [9,29]	
Complement C3	MMSE, ADAS-cog13, diagnostic groups		Brain amyloid burden [14], hippocampal volume [11], plaque clearance in mouse model [23], diagnostic groups [6]
Vitronectin	CSF AB without MTC, diagnostic groups		Diagnostic groups [6]
von Willebrand factor		Diagnostic groups [31]	
Cortisol	Volume of the left and right hippocampi, MMSE, diagnostic groups	Diagnostic groups [9]	Diagnostic groups [25,26]
Serum amyloid p-component			'AD like' pathology in vitro [32], diagnostic groups [30]
AXL receptor tyrosine kinase			
Interleukin-3	Diagnostic groups		Diagnostic groups [27]
Interleukin-13			
Matrix metalloproteinase-9 total		Diagnostic groups [9]	Breakdown of the blood brain barrier [37], diagnostic groups [28]
APOE	Diagnostic groups	Diagnostic groups [9]	Breakdown of the blood brain barrier [37]
Leptin	CSF AB, thickness of the right entorhinal cortex, diagnostic groups		
Immunoglobulin E (IgE)			
